# Social cognitive training among individuals with schizophrenia: Identifying responders to treatment^☆^

**DOI:** 10.1016/j.scog.2025.100350

**Published:** 2025-02-12

**Authors:** Anja Vaskinn, André Løvgren, Ole A. Andreassen, Kjetil Sundet

**Affiliations:** a Centre for Research and Education in Forensic Psychiatry, Oslo University Hospital, Norway; b Centre for Precision Psychiatry, Faculty of Medicine, University of Oslo, Norway; c Section for Psychiatric Treatment Research, Oslo University Hospital, Norway; d Section for Precision Psychiatry, Oslo University Hospital, Norway; e Department of Psychology, University of Oslo, Norway

**Keywords:** Social cognition, Emotion perception, Interventions, Reliable change index, Cognitive remediation

## Abstract

In this follow-up study of a previous randomized controlled trial of targeted facial affect recognition training among individuals with schizophrenia, reliable change indices (RCIs) were employed to identify responders to treatment. The original study found improved theory of mind at 3-month follow-up. The current study included 15 participants who received the intervention and who completed all three assessment points in the original study. Six of them had RCIs over the cutoff (≥+1.64), indicating that they had a clinically meaningful and statistically reliable improvement in ToM. The responders had significantly higher psychotic symptom level at baseline, but no other group differences between responders and nonresponders were identified. The study found no support for suggested moderators of treatment effect of social cognitive training (sex, education). As no consistently replicated barriers to treatment gains have been identified, we suggest that social cognitive training, where available, should be offered to interested clients.

## 1 Introduction

Social cognitive training (SCT) is a psychosocial treatment intervention targeting impairments in social cognition. Social cognition involves different domains, such as emotion processing, mentalizing/theory of mind (ToM), attributional bias/style, and social perception (Green et al., 2019). Impairments in all domains are present in individuals diagnosed with schizophrenia (Weinreb et al., 2022). The impairments show longitudinal stability (McCleery et al., 2016) and do not respond to antipsychotic medication (Kucharska-Pietura and Mortimer, 2013). The social cognitive impairments are a particularly robust predictor of functioning (Halverson et al., 2019), and therefore, arguably, among the most important characteristics of the disorder. This is reflected in the inclusion of social cognition in the description of schizophrenia in the latest editions of the two diagnostic manuals, DSM-5 and ICD-11. The functional consequences of the social cognitive impairments have led to the development of a number of interventions that target these difficulties, with an ultimate goal of improving the lives of people with schizophrenia.

Overall, we know that SCT is beneficial for individuals with schizophrenia. Although transfer effects to daily life have been difficult to achieve, several meta-analyses show moderate-sized improvement in emotion perception, ToM and social perception (Nijman et al., 2020; Yeo et al., 2022). Based on such evidence, the most recent version of the European Psychiatric Association's guidelines for treatment of cognitive impairment in schizophrenia (Vita et al., 2022) recommends SCT for the treatment of social cognitive impairments.

In spite of overall positive effects, the evidence suggests that not everybody benefits from the treatment to the same extent. Meta-analyses imply both treatment-related (external) and participant-related (internal) moderators of treatment effect. The latter include birth-assigned sex and length of education, in that SCT appears to provide stronger effects for females and for individuals with less education (Nijman et al., 2020; Yeo et al., 2022).

An alternative to moderator analyses for assessing variability is to identify and describe individuals that reliably improve after SCT. This is important, given the inter-individual variability in social cognitive performance among persons with schizophrenia that has been identified (Rocca et al., 2016; Hajdúk et al., 2018; Vaskinn et al., 2022). From this variability follows a likelihood of variability also in response to SCT. To our knowledge, this individual-level alternative to moderator analyses has thus far not been undertaken for individuals with schizophrenia who have received SCT. For cognitive remediation, some studies have applied reliable change indices (RCIs) to study treatment response on the individual level (Medalia and Richardson, 2005; Cellard et al., 2016). RCIs indicate whether a change is clinically meaningful and statistically reliable. They have a long history in the neuropsychological literature, including in longitudinal cognitive studies in schizophrenia (Harvey et al., 2010; Gray et al., 2014; Flaaten et al., 2022), when there is a need to show that improvement is not only due the practice effects of repeated testing. RCIs are clearly of value also in cognitive intervention studies.

In a previous randomized controlled trial (RCT) of targeted facial affect recognition training (Vaskinn et al., 2019), we found that ToM was improved at 3-month follow-up, providing support for the generalizability and durability of SCT. Most likely, however, there was variability in response to treatment among the 24 individuals that received the intervention. The aim of the current follow-up study is to identify and describe individuals who responded to SCT.

## 2 Methods

### 2.1 Participants

In this follow-up study, only those participants that received the intervention in our original RCT (Vaskinn et al., 2019) and who completed all three assessments (baseline T_1_, post-treatment T_2_, 3-month follow-up T_3_), were included (*n* = 15).

### 2.2 Measures

The current follow-up study included both clinical and cognitive measures. We report data for clinical symptoms assessed with the Positive and Negative Syndrome Scale (PANSS; Kay et al., 1987) and the Global Assessment of Functioning-symptoms (GAF-s: Pedersen et al., 2007). IQ was measured with the 2-test version of Wechsler Abbreviated Scale of Intelligence (WASI: Wechsler, 2007). Four social cognition measures from three theoretical social cognitive domains were used. Pictures of Facial Affect (PFA: Frommann et al., 2002) and Emotion in Biological Motion (EmoBio: Heberlein et al., 2004) assess *emotion perception*, from faces and bodies, respectively. *Social perception* was measured with a short version of the Relationships Across Domains test (RAD: Vaskinn et al., 2017). A *ToM* measure, i.e. the Movie for the Assessment of Social Cognition (MASC: Dziobek et al., 2006), also formed part of the protocol. In addition, demographic variables (age, sex, years of education) and illness duration were examined.

### 2.3 Reliable change index

For the identification of responders, we calculated a reliable change index (RCI) from baseline (T_1_) to follow-up assessment (T_3_) for the ToM measure (MASC), the outcome measure which was significantly improved in the original study. The RCI was based on Christensen and Mendoza's (1986) early formula:

$$\mathrm{RCI}=\frac{x_{2}-x_{1}}{S_{\text{diff}}}$$

where S_diff_ = standard error of difference between two test scores (standard error of measurement of the difference). Practice effects were handled by changing the numerator, taking the control group into consideration (Duff, 2012):

$$\mathrm{RCI}=\frac{\left(T_{3}–T_{1}\right)–\left(M_{3}–M_{1}\right)}{S_{\text{diff}}}$$

T_1_ = mean score of the intervention group at baseline, T_3_ = mean score of intervention group at 3-month follow-up, M_1_ = mean score of control group at baseline, M_3_ = mean score of control group at 3-month follow-up.

S_diff_ was calculated using the standard deviation of the M_1_ and M_2_ scores (i.e., in the control group), as well as the correlation between M_1_ and M_2_ (r_12_). M_2_ is the score of the control group at the second assessment, immediately after the training. It was chosen over the M_3_ score because S_diff_ is concerned with a measure's reliability, not with matching the exact assessment point with that of the group of interest (i.e., T_3_).

$$S_{\text{diff}}=\sqrt{{{\mathrm{SEM}}_{1}}^{2}+{{\mathrm{SEM}}_{2}}^{2}}$$

${\mathrm{SEM}}_{1}=\mathrm{SD}\sqrt{1-r_{12}}$ where SD is the standard deviation at M_1_ (i.e., in the control group). ${\mathrm{SEM}}_{2}=\mathrm{SD}\sqrt{1-r_{12}}$ where SD is the standard deviation at M_2_ (i.e., in the control group).

This formula for calculating the S_diff_ has been recommended for cognitive test scores (Iverson et al., 2003) and has been shown to produce satisfactory results compared to other formulas (Hinton-Bayre, 2011). We used a 90 % confidence interval, so that RCIs ≥+1.64 were considered to indicate a reliable improvement.

### 2.4 Statistical analyses

Responders were compared to nonresponders on demographic and baseline clinical and (non)social cognitive variables, using independent samples *t*-tests with Cohen's *d* effect sizes.

## 3 Results

**Fig. 1:**
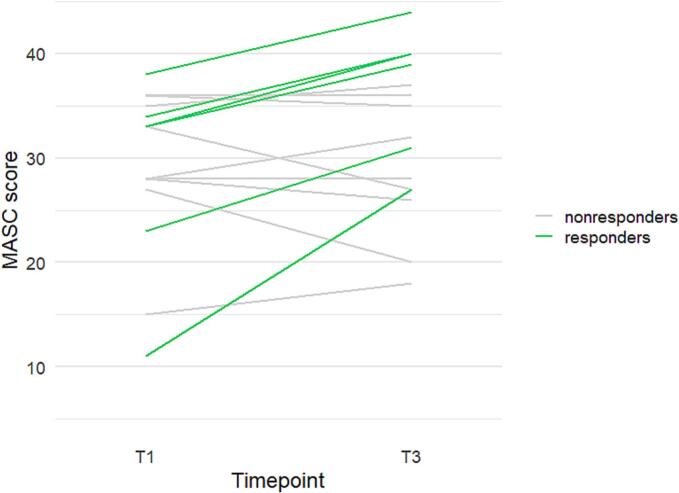
Individual MASC trajectories from pre-treatment baseline assessment (T1) to 3-month follow-up assessment (T3) of the 15 individuals that completed the intervention. MASC = Movie for the Assessment of Social Cognition.

Six of the 15 individuals that completed the intervention were characterized as responders, i.e. they had a RCI ≥+1.64. The RCIs for all participants can be seen in Table 1. The individual MASC slopes from pre-treatment baseline assessment (T_1_) to 3-month follow-up assessment (T_3_) are provided in Fig. 1. Comparisons between responders and nonresponders are reported in Table 2. Responders had a significantly higher positive symptom load at baseline. No other group differences were statistically significant, but the difference for emotion perception was medium-sized for body emotion perception (EmoBio) and large for facial emotion perception (PFA).

**Table 1:** Reliable change index (RCI) for MASC scores of the 15 individuals that completed the intervention.

MASC RCI	Number of participants
No reliable change: Nonresponders (*n* = 9)	
−1.11	1
−0.87	1
0.11	1
0.36	1
0.60	2
1.09	1
1.34	1
1.59	1

Reliable change: Responders (*n* = 6)	
2.08	3
2.32	1
2.57	1
4.53	1

MASC = Movie for the Assessment of Social Cognition.

**Table 2:** Comparison of responders and nonresponders among the 15 individuals that completed the intervention.

	Responders n = 6	Nonresponders n = 9	Statistic	Effect size/Cohen's *d*
Demographics				
Age	33.2 (11.5)	29.9 (8.7)	*t* = 0.63	0.33
Sex	5/1	8/1	χ^2^ = 0.10	φ = 0.08
WASI IQ	108.5 (9.4)	105.0 (12.5)	*t* = 0.58	0.31
Years of education	12.7 (2.9)	12.2 (1.9)	*t* = 0.37	0.19

Clinical characteristics at baseline				
Illness duration	7.7 (10.5)	5.7 (6.6)	*t* = 0.46	0.24
GAF-s	44.0 (11.9)	46.3 (11.6)	*t* = 0.38	0.20
PANSS positive symptoms	18.0 (6.9)	12.4 (4.5)	*t* = 1.91*	1.01
PANSS negative symptoms	15.5 (3.5)	16.3 (5.7)	*t* = 0.32	0.17

Social cognition at baseline				
PFA	86.2 (9.5)	76.1 (11.7)	*t* = 1.75	0.92
EmoBio	0.85 (0.09)	0.80 (0.10)	*t* = 1.07	0.57
RAD	24.5 (2.7)	24.9 (5.4)	*t* = 0.16	0.09
MASC	28.7 (10.0)	29.6 (6.6)	*t* = 0.21	0.11

WASI = Wechsler Abbreviated Scale of Intelligence; GAF = Global Assessment of Functioning-symptoms; PANSS = Positive and Negative Syndrome Scale; PFA = Pictures of Facial Affect; EmoBio = Emotion in Biological Motion; RAD = Relationships Across Domains; MASC = Movie for the Assessment of Social Cognition.

## 4 Discussion

This study found that six of 15 persons with schizophrenia that underwent facial affect recognition training (40 %) had a clinically significant and reliable improvement in ToM. Two other participants probably also benefited, but their RCIs did not quite reach the cutoff. Two participants appear to have deteriorated, but not in a clinically significant way. Our results confirm heterogeneity in response to SCT, as expected based on studies showing social cognitive heterogeneity (Rocca et al., 2016; Hajdúk et al., 2018).

Responders to facial affect recognition training had a higher psychotic symptom level at baseline. The symptom level was, however, not severe, but corresponded to, on average, “minimal” to “mild” psychotic symptoms. Thus, this finding should not to be taken as an indication that psychosis is an advantage when undergoing SCT. On the other hand, a certain level of emotion perception may be advantageous when undergoing SCT focusing on this domain, given the better baseline emotion perception performance among responders compared to nonresponders (medium-large effect sizes). Responders were slightly older and had somewhat higher IQ than nonresponders (small effect sizes), but other group differences were negligible.

There were no sex differences compared to nonresponders, and the six individuals who responded to treatment showed variability in education (two had master's degrees, one had only completed the compulsory 9 years of schooling). In other words, we did not find support for the suggested moderators of treatment effect (birth-assigned sex, education) (Nijman et al., 2020; Yeo et al., 2022).

Although the responders differed somewhat from nonresponders for age and current IQ, there was substantial in-group variation. Specifically, responders showed variability for age (two in their early 20ies, 2 in their 30ies, 2 in their 40ies), and current IQ (3 on par with healthy control participants, 2 at the schizophrenia mean). This indicates that an individual should not be refused SCT based on his or her demographic characteristics. Illness duration also varied widely, with two having been ill with schizophrenia for more than 15 years, and the other four from 1 to 2 years.

In sum, our results suggest that SCT may be beneficial for individuals with schizophrenia regardless of their age, birth-assigned sex and illness stage. The study provides no indication that SCT is a harmful intervention, given that nobody experienced a clinically significant deterioration. We must stress that our study was small and that caution is needed when interpreting the findings. However, we would argue that SCT could be offered to everyone with a diagnosis of schizophrenia, since no consistent individual treatment barriers have been consistently demonstrated. This is in line with the suggestions of the European Psychiatric Association in their treatment guidelines for cognitive remediation, including SCT (Vita et al., 2022); where they point to the inconsistency in moderators of treatment effect between studies. On a final note, we acknowledge that the findings of our study, undertaken in Oslo, Norway, may not generalize to other cultural settings.

To conclude, no barriers to treatment gains have been robustly identified. Current evidence does not support selection of participants for SCT based on individual characteristics. In clinical practice, where available, SCT should be offered to interested clients.

## CRediT authorship contribution statement

**Anja Vaskinn:** Writing – review & editing, Writing – original draft, Visualization, Resources, Project administration, Methodology, Investigation, Funding acquisition, Formal analysis, Data curation, Conceptualization. **André Løvgren:** Writing – review & editing, Methodology, Investigation, Funding acquisition, Conceptualization. **Ole A. Andreassen:** Writing – review & editing, Resources. **Kjetil Sundet:** Writing – review & editing, Resources, Methodology, Funding acquisition, Conceptualization.

## Funding

The study received funding from the Norwegian Extra-Foundation for Health and Rehabilitation/Norwegian Council for Mental Health (Grant #2011/3/0025 to AV), the 10.13039/501100006095South-Eastern Norway Regional Health Authority (Grant #2010007 and #2017069 to AV), Division of Mental Health and Addiction, Oslo University Hospital (to AV and AL), and the Department of Psychology, 10.13039/501100005366University of Oslo (personal postdoctoral grant to AV), the Fulbright Foundation for Educational Exchange (visiting researcher scholarship to AV).

## Declaration of competing interest

OAA is a consultant to Cortechs.ai and Precision Health, and has received speaker's honorarium from Lilly, Lundbeck, Janssen and Otsuko. All other authors declare that they have no competing interests.
